# A Hand-Book of Diseases of the Eye and Their Treatment

**Published:** 1893-06

**Authors:** 


A HAND-BOOK OF DISEASES OF THE EYE AND THEIR TREATMENT, by Henry R. Swanzy, A.M., M.B., F.R.C.S.I., Surgeon to the National Eye and Ear Infirmary and Ophthalmic Surgeon to the Adelaide Hospital, Dublin, etc. Fourth Edition. With Illustrations. Philadelphia: P. Blakiston, Son & Co. 1892. Price, $3.

In its fourth edition, this little book seems to be an epitome of all that can be said, and said well in a book of its size. Its subject matter is brought squarely up to the times in all respects. In saying all that the author should say in a handbook and not all that he might say, only shows the good common sense of him who prepared this magnificent little book to serve the purpose that he intended.

This edition is, in many ways, an improvement upon the third edition, which we regarded as a most excellent book for the general practitioner, or even for the special worker in eye diseases. As an epitome of all the diseases of the eye, not only in the etiology, pathology, and symptology of these diseases, but in the short and concise delineation of their treatment the little book is admirable. We would commend it in most all, if not quite all, of its precepts, even in its sometimes seemingly dictatorial forms of treatment.

Altogether, the little book is admirable, and nonc better has been issued in the form of a handbook in the last decade.

